# Chief Executive Officers Entrepreneurial Orientation, Dynamic Capabilities, and Firm Performance: The Moderating Effect of the Manufacturing Industry

**DOI:** 10.3389/fpsyg.2021.707971

**Published:** 2021-09-24

**Authors:** Yueyue Liu, Meng Xi, Yingya Jia, Xiulin Geng

**Affiliations:** ^1^School of Business, Nanjing University, Nanjing, China; ^2^School of Management, Shanghai University, Shanghai, China

**Keywords:** chief executive officers entrepreneurial orientation, firm performance, dynamic capabilities, the manufacturing industry, China context

## Abstract

This study explores the implications of CEO entrepreneurial orientation for firm performance through corporate dynamic capabilities. It explores the moderating effects of firm industry type on the above indirect effect. Based on 188 matched sample data collected from vice chief executive officers (CEOs) of Chinese firms, this study found that CEO entrepreneurial orientation was positively related to corporate dynamic capabilities and firm performance and that corporate dynamic capabilities mediated the positive relationship between CEO entrepreneurial orientation and firm performance. Firm industry type moderated the direct effect of CEO entrepreneurial orientation on corporate dynamic capability, and the indirect effect of CEO entrepreneurial orientation on firm performance through corporate dynamic capability. Both direct and indirect effects were stronger in manufacturing enterprises. The findings enrich the CEO entrepreneurial orientation literature by extending the existing knowledge on its underlying mechanism and its impact on firm performance, as well as its boundary conditions.

## Introduction

The relationship between firm-level entrepreneurial orientation and firm performance has been examined by many theoretical and empirical studies (Higgs and Rowland, 2005; Covin et al., 2006; Rauch et al., 2009; Keil et al., 2017; Shen et al., 2021). The findings over the last three decades have indicated that the positive relationship between both variables has been supported widely (*r*=0.242, see, e.g., Rauch et al., 2009 for a review) and is robust to different cultural contexts (Rauch et al., 2009; Saeed et al., 2014). Some scholars have shifted to an emerging stream in the entrepreneurial orientation literature, namely, individual – for example, chief executive officer (CEO) – entrepreneurial orientation, and have explored its consequences (Bolton, 2012; Keil et al., 2017; Liu and Xi, 2021; Wang et al., 2021; Zhang et al., 2021). The value of studying individual entrepreneurial orientation lies in the fact that firm entrepreneurial orientation is implemented and strengthened by individuals including firm owners, top- and mid-level managers, and individual employees (Hughes et al., 2018) and manifests at all organizational levels (Wales et al., 2011; Wiklund and Shepherd, 2011; Covin et al., 2020). Individual entrepreneurial orientation is a more proximal reflection of the operation of firm entrepreneurial orientation that can be linked to firm outcomes.

We make an inroad into the emerging stream of CEO entrepreneurial orientation literature. The CEO of a firm is the key decision maker and is ultimately responsible for its organizational outcomes (Hambrick and Mason, 1984; Hambrick and Finkelstein, 1987; Hambrick, 2007; Xi et al., 2017; Li et al., 2021). CEOs’ attention, emphasis, and openness to entrepreneurial activities and behaviors may affect firm performance (Hambrick, 2007; Keil et al., 2017; Liu and Xi, 2021). To further contribute to the performance implication of CEO entrepreneurial orientation, the first aim of this study is to investigate how the CEO’s attention, emphasis, and openness to entrepreneurial activities affect firm performance.

Then, we explored the mediating effect of dynamic capabilities in the relationship between CEO entrepreneurial orientation and firm performance. We focused on dynamic capabilities because of their entrepreneurial ability to adapt to a rapidly changing and uncertain organizational environment (Teece, 2007; Li and Liu, 2014). Therefore, CEO entrepreneurial orientation with innovativeness, proactiveness, and risk-taking characteristics may have a great impact on the development of dynamic capabilities in an uncertain environment (Lawson and Samson, 2001; Jiao et al., 2010; Haarhaus and Liening, 2020). Entrepreneurship can promote the circulation, dissemination, and transfer of knowledge within and between organizations and ultimately develop dynamic capabilities (Zahra et al., 1999). Helfat and Martin (2015) stated that enterprises need to quickly identify and understand environmental changes and have the dynamic capabilities to adapt to such changes in order to gain competitive advantage in market competition. The establishment of dynamic capabilities is both a key factor for an enterprise to cope with the uncertainties of the external environment and gain competitive advantage, and an important prerequisite for firms to improve their performance (Jiao et al., 2010; Zeng and Song, 2011).

Furthermore, this paper probes into the possible boundary condition that amplifies or narrows the influence of CEO entrepreneurial orientation. The manufacturing industry plays a crucial role in the adjustment of the economic structure in China (Pan and Zhao, 2019; Shen et al., 2020), which is our empirical context. In the course of industrial reforms, the transformation and upgrading of manufacturing enterprises face more severe challenges and uncertainties (Cheng and Song, 2016). It is an inevitable choice for manufacturing enterprises to enhance their connotation development, to strengthen their dynamic capacity, and to promote enterprise innovation. Mao and Wang (2015) pointed out that giving full play to entrepreneurship and enhancing organizational ability are important ways to improve innovation and firm performance of manufacturing enterprises. In the manufacturing industry, the CEO’s attention to innovativeness and proactiveness intends to help the firm better deal with challenges and grasp opportunities emerging from industrial reforms, thus improving firm dynamic capabilities and performance. However, the existing literature has rarely treated the industry types of firms, such as manufacturing and non-manufacturing, as an important variable. Therefore, it is necessary to directly discuss the influence of the enterprise industry type on the variables of interest, in order to increase our differentiated understanding of the influence mechanism of CEO entrepreneurial orientation on firm performance in manufacturing and non-manufacturing enterprises. In this study, we will further examine whether firm industry type (manufacturing vs. non-manufacturing) shapes the positive relationship between CEO entrepreneurial orientation and dynamic capability, and the indirect effect of CEO entrepreneurial orientation and firm performance through dynamic capability.

Our work makes several important contributions to the entrepreneurial orientation literature. First, we seek to open up the “black box” of the implications of CEO entrepreneurial orientation for firm performance. Although CEO entrepreneurial orientation has been found to “matter” for firm value creation (Keil et al., 2017), we found that it can be a facilitator for firm performance, too. Second, this study is among the first to investigate how CEO entrepreneurial orientation improves firm performance by promoting the dynamic capabilities of the firm, which expands the underlying mechanism of entrepreneurial orientation on firm performance (Liu and Xi, 2021). Third, this study tests the moderating effect of industry type, instead of the constantly used firm size (Ha-Brookshire, 2009; Chelliah et al., 2010) and ownership (Campbell et al., 2010; Zahra, 2012), on the relationship between CEO entrepreneurial orientation and firm dynamic capabilities. Compared with non-manufacturing firms, CEO entrepreneurial orientation in manufacturing firms has a stronger impact on dynamic capabilities, which enhances our understanding of the boundary conditions for the impact of CEO entrepreneurial orientation on firm dynamic capabilities. This study provides a theoretical basis and empirical reference for future research on the manufacturing industry as a moderating variable, especially in the Chinese context.

The rest of this study is organized as follows. First, we present the theoretical development and hypotheses. Next, we present our research method and statistical results. Finally, we present a discussion that also details the theoretical and practical implications, limitations, and directions for future research.

## Theoretical Framework and Hypotheses

**Figure 1:**
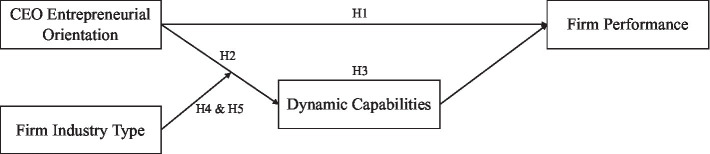
The research framework of this study.

In this section, we trace the development of our overall research model by first exploring the main effect of CEO entrepreneurial orientation and firm performance and then the mediating role of dynamic capabilities. We further incorporate industry type as a moderating variable to help explain the extent to which CEO entrepreneurial orientation influences firm performance through dynamic capabilities. Figure 1 shows the hypothesized model of this study.

### CEO Entrepreneurial Orientation and Firm Performance

Entrepreneurship orientation (EO), generally conceived as a firm-level construct, refers to “an organizational attribute reflecting how ‘being entrepreneurial’ is manifested in organizations or business units” (Covin and Wales, 2019: 4) and comprises innovativeness, proactiveness, and risk taking (Miller, 1983; Covin and Slevin, 1989; Rauch et al., 2009). However, CEOs, as key decision makers, are crucial to firms’ strategic activities, decision making, and organizational outcomes (Hambrick and Mason, 1984; Hambrick, 2007; Busenbark et al., 2016; Yuan et al., 2017; Bao et al., 2020; Liu et al., 2021). A CEO can have power over strategic choices, investment decisions, entrepreneurial activities, and management practices (Jensen and Murphy, 1990; Bertrand and Schoar, 2003; Hambrick and Quigley, 2014) and influence over everyone in the company (Keil et al., 2017). This influence manifests in their behaviors, expectations, and in signaling the behaviors that are considered desired within the organization (Keil et al., 2017). Thus, firm-wide actions and behaviors instigated by entrepreneurial orientation and their performance consequences are critically influenced by CEOs (Cho and Hambrick, 2006; Simsek et al., 2015; Keil et al., 2017).

Liu and Xi (2021) defined CEO entrepreneurial orientation as “the extent to which the CEO is inclined to emphasize innovation and invest in innovative activities to achieve a competitive advantage, proactively cope with competition and identify business opportunities, and take business-related risks without fear of failure” (p. 3). According to this definition, CEO entrepreneurial orientation reflects the CEOs’ attention toward, openness to, and emphasis on entrepreneurial behaviors and activities, demonstrating their commitment to entrepreneurial orientation (Ocasio, 1997, 2011; Keil et al., 2017; Wales et al., 2020; Liu and Xi, 2021). Given the key role of a CEO in an organization (Hambrick, 2007), and the positive relationship between entrepreneurial orientation and firm performance (Rauch et al., 2009; Saeed et al., 2014), we expect CEO entrepreneurial orientation to have a positive impact on firm performance.

First, by devoting considerable and consistent attention to entrepreneurship, the CEO emphasizes the production, adoption, and implementation of useful and novel ideas, products, and/or procedures (Farr and Ford, 1990) through various organizational channels (Ocasio, 1997). Research suggests that the effort and time spent on anticipating demand and promoting new products or services can often lead to higher performance (Ireland et al., 2003; Rauch et al., 2009).

Second, considering that pursing new value-creating entrepreneurial opportunities is important to lead rather than follow competitors in a certain business area (Covin and Slevin, 1989), CEOs with high entrepreneurial orientation are more likely to constantly seek out new and value-creating entrepreneurial opportunities (Lumpkin and Dess, 1996; Sullivan, 2010; Keil et al., 2017) by allocating their time and deploying firm resources (Keil et al., 2017).

Third, CEOs’ attention, emphasis, and openness to entrepreneurial behaviors are a signal to invoke organizational members’ innovative and pioneering behaviors. A CEO’s entrepreneurial orientation can be distributed through structural channels within the organization, thus encouraging engagement in value-creating entrepreneurial activities (Ling et al., 2008; Keil et al., 2017; Yuan et al., 2017), which may be conducive to both establishing new sources of profit growth and achieving success in the future.

Finally, by focusing their attention on entrepreneurial behaviors and activities, CEOs can directly and indirectly shape organizational members’ perceptions, attitudes, behaviors, and performance consequences (Salancik and Pfeffer, 1978; Ocasio, 1997, 2011; Keil et al., 2017).

In sum, by demonstrating attention, emphasis, and openness to entrepreneurial behaviors and activities, CEOs may evoke a firm-wide entrepreneurial orientation (Chatterjee and Hambrick, 2007; Simsek et al., 2010; Keil et al., 2017; Liu and Xi, 2021) that can enhance firm performance (Covin and Slevin, 1986; Wiklund and Shepherd, 2003; Dimitratos et al., 2004; Rauch et al., 2009). Keil et al. (2017) found that CEO entrepreneurial orientation positively results in firm value creation. Therefore, CEO entrepreneurial orientation can be seen as a factor fostering firm performance. We propose the following:

*Hypothesis 1*: CEO entrepreneurial orientation is positively related to firm performance.

### The Mediating Role of Dynamic Capabilities

With the rapid development of science and technology and the fiercely growing market competition in recent years, some authors have argued that the resource-based theory does not fully explain how companies gain competitive advantage in a rapidly changing business environment and have emphasized the role and importance of dynamic capabilities (Teece et al., 1997; Eisenhart and Martin, 2000; Teece and Pisano, 1994; Winter, 2003). To gain competitive advantage in a rapidly changing environment, a firm’s capabilities must be dynamic (Teece et al., 1997; Teece, 2007; Ren et al., 2021). It must have the ability to constantly update its capabilities in order to ensure coordination between the external environment and firm strategy (Li and Liu, 2014; Teece and Leih, 2016).

Studies have identified dynamic capabilities as a source of sustainable competitive advantage (Teece et al., 1997; Eisenhart and Martin, 2000). Teece et al. (1997) defined dynamic capabilities as the capabilities of a firm to integrate, develop, and reconfigure internal and external competences in order to respond quickly to a changing business environment. Teece (2007) proposed a sensing-seizing-reconfiguring framework of dynamic capabilities, which center on sensing and seizing opportunities, managing threats, and reconfiguration. We argue that CEO entrepreneurial orientation can effectively enhance these three capabilities.

First, environmental scanning is an important part of identifying opportunities and predicting competitive threats (Peteraf and Bergen, 2003). High environmental scanning capability means that a firm can quickly and effectively identify entrepreneurial opportunities and threats in a changing environment. Highly entrepreneurially oriented CEOs pursue new value-creating entrepreneurial opportunities by engaging in meaning-creating activities in uncertain environments (Hill and Levenhagen, 1995; Weick, 1995; Alvarez and Barney, 2007). Jantunen et al. (2005) found that higher levels of entrepreneurial orientation support the ability to identify opportunities, which, in turn, have a positive impact on dynamic capabilities. Thus, CEO entrepreneurial orientation can enhance the capability of sensing opportunities.

Second, CEOs with high entrepreneurial orientation emphasize the production, adoption, and implementation of useful and novel ideas, products, and procedures (Farr and Ford, 1990), which helps companies create, define, identify, and take advantage of new market opportunities before their competitors. Wiklund (1999) found that entrepreneurial orientation has a significant impact on the improvement of organizational culture, learning, and ability. Thus, entrepreneurial strategy is a key choice in dealing with complex environments and managing transformation (Zahra et al., 2006). By paying attention to, laying emphasis on, and exhibiting openness toward entrepreneurial behaviors and activities, and demonstrating a commitment to entrepreneurial orientation, CEOs divide their time, energy, and resources between entrepreneurial activities and actions (Keil et al., 2017). Thus, CEO entrepreneurial orientation enhances their ability to seize opportunities.

Finally, CEOs with high entrepreneurial orientation emphasize the introduction of creative ideas, as well as new methods and procedures, and have high tolerance for change, which helps their organizations adapt better. Sirmon and Hitt (2003) posited that CEOs are the core agents for change. When an organization’s external environment changes dramatically, the CEO leads the top management to activate the dynamic capabilities that are embedded in the organization, strip the redundant resources that cannot adapt to the complex environment, and reconfigure the existing resources to develop capabilities in order to adapt to the enterprise’s existing and new markets. Research has found a positive relationship between entrepreneurial orientation and dynamic capabilities (SubbaNarasimha, 2001; Jiao et al., 2010; Monteiro et al., 2017). Thus, CEO entrepreneurial orientation enhances the abilities to manage threats and engage in reconfiguration. Accordingly, we hypothesize as follows:

*Hypothesis 2*: CEO entrepreneurial orientation is positively related to dynamic capabilities.

Teece et al. (1997) defined dynamic capability as the ability to investigate how an enterprise reconfigures internal and external resources through a series of behaviors, so that the enterprise can quickly adapt to environmental change, achieve competitive advantage and gain profits, and improve organizational performance. Eisenhart and Martin (2000) argued that dynamic capabilities serve as a means to change the resource base and help form new value creation strategies and obtain competitive advantage.

To explain how dynamic capabilities promote and achieve competitive advantage and firm performance, scholars have explored the operational mechanism of dynamic capabilities (Zahra and George, 2002; Helfat and Peteraf, 2003). For instance, such capabilities can improve existing business activities by selecting technical knowledge pertaining to an existing knowledge base during the evolution of business and operational technology models (Eisenhart and Martin, 2000; Zollo and Winter, 2002; Teece, 2010). Thus, dynamic capabilities enable companies to achieve outstanding performance in the long term (Teece, 2007; Wilden et al., 2013; Pezeshkan et al., 2016). Some empirical studies have supported the positive impact of dynamic capabilities on firm performance (Prange and Verdier, 2011; Protogerou et al., 2012; Lin and Wu, 2014; Fainshmidt et al., 2016; Pezeshkan et al., 2016; Zhou et al., 2021). For example, in a meta-analysis, Fainshmidt et al. (2016) found that dynamic capabilities were positively related to firm performance and that the positive relationship was stronger in industries with higher levels of technological dynamism. Lin and Wu (2014) found that both firm dynamic learning and reconfiguration capabilities were positively related to firm performance.

Considering the positive impacts of CEO entrepreneurial orientation on both firm performance (Hypothesis 1) and dynamic capabilities (Hypothesis 2), this study expects the latter to play a mediating role in the relationship between CEO entrepreneurial orientation and firm performance. That is, the positive effect of CEO entrepreneurial orientation on firm performance is achieved through the enhancement of dynamic capabilities. Therefore, this study proposes the following hypothesis:

*Hypothesis 3*: Dynamic capabilities mediate the relationship between CEO entrepreneurial orientation and firm performance.

### The Moderating Role of the Manufacturing Industry

Compared with the number of studies that have examined the moderating effect of firm ownership (Campbell et al., 2010; Zahra, 2012) and size (Ha-Brookshire, 2009; Chelliah et al., 2010), only a few have explored the moderating effect of firm industry type. This study considers it is necessary to specifically discuss the industry type of enterprises in the current stage, such as manufacturing and non-manufacturing industry, which may have a moderating effect on the relationship between CEO entrepreneurial orientation and dynamic capabilities. This is because, first, the manufacturing industry is the foundation of China’s economic development and plays a vital role in the strategic adjustment of economic structure (Pan and Zhao, 2019). Second, China’s manufacturing industry is at a critical stage of transformation and upgrading from “Made in China” to “Create in China.” Compared with the industrial transformation and upgrading of non-manufacturing enterprises, the transformation and upgrading of the manufacturing industry encounter more severe challenges (Cheng and Song, 2016). Third, compared with non-manufacturing enterprises, manufacturing ones pay more attention to labor and property costs. With the gradual loss of China’s demographic dividend and land cost advantage (Jin, 2015), manufacturing enterprises must undergo transformation and upgrading, enhance connotation development, and strengthening their dynamic capacity building approaches. Fourth, China’s manufacturing industry has already formed a complete supply chain and industrial supporting system, with low prices and fast delivery speeds in order to ensure the smooth operation of the global value chain.

However, with the rapid development of information technology and the constant changes in the market environment, China’s manufacturing enterprises continue face increasing environmental uncertainties (Yu et al., 2020). CEOs with high entrepreneurial orientation are more likely to play an important role in this context (Mao and Wang, 2015). As for the moderating effect of the manufacturing industry in the relationship between CEO entrepreneurial orientation and dynamic capabilities, we argue that there is a stronger positive relationship between CEO entrepreneurial orientation and dynamic capabilities in manufacturing enterprises for the following reasons.

First, compared with non-manufacturing enterprises, manufacturing ones, especially the labor-intensive kind, need to strengthen the construction of enterprise dynamic capabilities in order to improve their competitiveness (Geng and Yuan, 2010). Therefore, if the CEOs of manufacturing enterprises demonstrate the pursuit of innovation, take the initiative, and dare to take risks in daily behaviors like decision making, management, and leadership, it will better meet the needs of manufacturing enterprises to strengthen their dynamic capacities.

Second, compared with non-manufacturing enterprises, manufacturing ones encounter greater uncertainties in market demand, material supply, competition, and new product technology. Leaders of manufacturing enterprises need to adopt flexible development strategies to remain consistent with the external environment, in order to ensure high profits and sales performance (Chang et al., 2002; Singh et al., 2013). When the CEO of manufacturing enterprises has a high degree of entrepreneurial orientation, it is easier to stimulate the inner capabilities of these enterprises to develop new markets, products, and technologies.

Third, for manufacturing organizations operating in increasingly uncertain environments and volatile markets, dynamic capabilities can be considered a major competitive weapon, providing organizations with the ability to quickly identify market opportunities, manage threats, and respond to market competition (Singh et al., 2013). Therefore, manufacturing enterprises may pay more attention to the construction of enterprise dynamic capabilities. When CEOs in manufacturing enterprises show high commitment to entrepreneurial orientation, the influence of CEO entrepreneurial orientation on the dynamic capabilities may be stronger.

Finally, compared with non-manufacturing enterprises, digital transformation is becoming important for manufacturing enterprises with the introduction of digital technology in production systems (Lin et al., 2020a). Digital transformation refers to the application of digital technology and is a key driver of manufacturing transformation (Lin et al., 2020b). Lin et al. (2020b) found that dynamic capabilities are essential in order to remain competitive in a rapidly changing industrial environment in the course of enterprise transformation. CEOs of manufacturing enterprises with high entrepreneurial orientation tend to emphasize on the introduction of new technologies, methods, and procedures, and simultaneously, on digital transformation and the construction of enterprise dynamic capabilities. In light of the above analysis, we propose as follows:

*Hypothesis 4*: Enterprise industry type moderates the positive relationship between CEO entrepreneurial orientation and dynamic capabilities: Compared with non-manufacturing enterprises, the positive relationship between CEO entrepreneurial orientation and dynamic capabilities is stronger in manufacturing enterprises.

Thus far, this study has proposed to examine the positive impact of CEO entrepreneurial orientation on firm performance and has proposed that dynamic capabilities play a mediating role between CEO entrepreneurial orientation and firm performance and that manufacturing industry type plays a moderating role between CEO entrepreneurial orientation and dynamic capabilities. There may be a potential theoretical hypothesis underlying these assumptions. That is, the manufacturing industry type may moderate the indirect effect of CEO entrepreneurial orientation on firm performance through dynamic capabilities. Therefore, this study proposes the following:

*Hypothesis 5*: Firm industry type moderates the indirect influence of CEO entrepreneurial orientation on firm performance through dynamic capabilities. This indirect effect is stronger and weaker among manufacturing and non-manufacturing firms, respectively.

## Materials and Methods

### Participants and Procedure

Considering that the research questions in this paper mainly involve variables at the firm level, we collect data at the firm level by surveying top executives. We employed a quantitative research approach and collected data from firms based on the convenience sampling method. We collected data from four economic and technological development zones in Guangdong, Anhui, Jiangsu, and Tianjin, in China from March to September 2017. To ensure the quality and reliability of the collected data, we asked officials of the Administrative Committee of these four zones to help administer the survey.

To rule out common method bias, we adopted the pairing method for data collection. We invited two vice presidents each from 300 firms to participate in our study. We asked one VP to rate the CEO entrepreneurial orientation, and the other to rate the dynamic capability and firm performance. Both of two VPs rated CEO demographics and firm characteristics. In all, 210 firms returned the questionnaires after filling them. After deleting the unmatched observations, we obtained 188 matched data. The final effective response rate was 0.937.

### Sample Characteristics

Of the 188 CEOs, 91.5% were male with an average age ranging between 45 and 50years. The average education level was master’s degree including MBA and EMBA. Their average tenure (months) was 60.79 (S.D.=56.94). Out of 188 firms, 102 were private firms; 30 were state-owned enterprises; 40 were foreign firms; 16 were others; 110 were small- and medium-sized enterprises; 100 were manufacturing firms; and 88 were non-manufacturing firms. The average established years were 15.53 (S.D.=13.59).

### Measures

#### CEO Entrepreneurial Orientation

We employed the 9-item scale developed by Liu and Xi (2021) to measure CEO entrepreneurial orientation including innovativeness, proactiveness, and risk taking. A sample item for innovativeness was “Our CEO emphasizes on finding innovative solutions to problems.” A sample item for proactiveness was “In the face of market competition, our CEO emphasizes on being proactive rather than reactive.” A sample item for risk taking was “Our CEO is willing to take higher risks in pursuit of higher returns.” Vice presidents were asked to rate CEO entrepreneurial orientation based on a 7-point Likert scale ranging from 1 (strongly disagree) to 7 (strongly agree). The Cronbach’s coefficient of CEO entrepreneurial orientation was.937.

#### Corporate Dynamic Capability

Following previous studies (Helfat and Peteraf, 2003; Teece and Pisano, 1994; Jantunen et al., 2005; Jiao et al., 2010), we employed a 14-item scale to measure corporate dynamic capabilities. A sample item was “Our company has a rapid organizational response to market changes.” Vice CEOs were asked to rate dynamic capabilities based on a 7-point Likert scale ranging from 1 (strongly disagree) to 7 (strongly agree). The Cronbach’s coefficient of dynamic capabilities was 0.942.

#### Firm Performance

This study employed an 8-item scale developed by Katsikeas et al. (2006) to measure firm performance. Vice CEOs were asked to assess their firm’s performance relative to other firms in the industry based on a 7-point Likert scale that ranged from 1 (very low) to 7 (very high). These items pertained to sales, financial, and customer performance. The Cronbach’s coefficient of firm performance was 0.916.

#### Firm Industry Type

We asked vice presidents to report whether their companies were manufacturing firms. Accordingly, there were manufacturing (1) and non-manufacturing firms (0).

#### Control Variables

We controlled for the CEO’s demographic variables, including gender, age, education level, and tenure (by months), and firm characteristics including firm ownership structure (1=private and 0=others), firm size (1=small- or medium-sized enterprise with under 500 employees and 0=large-sized enterprise with over 500 employees), and established years that were found to correlate with firm performance (Hambrick and Mason, 1984; Hambrick and Finkelstein, 1987; Hambrick, 2007).

### Analytical Approach

Given that all the variables were collected at the firm level, we employed conditional process analysis in SPSS to test our hypotheses (Hayes, 2013).

## Results

Table 1 presents the means, standard deviations, and correlations of the variables. Consistent with our theoretical assessment, CEO entrepreneurial orientation was positively related to dynamic capability (*b*=0.552, *p*<0.01) and firm performance (*b*=0.382, *p*<0.01). Dynamic capability was positively related to firm performance (*b*=0.458, *p*<0.01).

**Table 1:** Descriptive statistics and correlations among the variables.

Variables	Mean	S.D.	1	2	3	4	5	6	7	8	9	10
1. CEO male	0.94	0.29										
2. CEO age	4.49	1.67	−0.024									
3. CEO education	4.01	1.26	0.121	−0.105								
4. CEO tenure	60.67	57.09	0.055	0.117	0.038							
5. Private	0.54	0.50	0.094	−0.192^**^	0.076	0.276^**^						
6. Manufacturing	0.53	0.50	−0.285^**^	0.199^**^	−0.401^**^	−0.165^*^	−0.262^**^					
7. Firm size	0.59	0.50	0.115	−0.103	0.180^*^	0.065	0.267^**^	−0.357^**^				
8. Firm age	15.53	13.63	0.069	0.131	−0.304^**^	−0.100	−0.270^**^	0.255^**^	−0.341^**^			
9. CEO EO	5.07	1.31	0.022	−0.284^**^	−0.110	0.130	0.096	−0.191^*^	−0.073	−0.151^*^		
10. Dynamic capabilities	5.80	0.66	0.036	−0.196^**^	−0.167^*^	0.116	−0.085	−0.083	−0.180^*^	−0.035	0.552^**^	
11. Firm performance	5.22	0.99	0.009	−0.211^**^	−0.039	0.097	0.086	−0.183^*^	−0.170^*^	−0.164^*^	0.382^**^	0.209^**^

*N=188; CEO, Chief Executive Officer; and EO, Entrepreneurial Orientation.*

* *p<0.05;*

** *p<0.01.*

### Hypothesis Testing

Table 2 presents the results of the regression analysis for dynamic capabilities and firm performance.

**Table 2:** Results of regression analysis for dynamic capabilities and firm performance.

Variables	Dynamic Capabilities		Firm Performance		
	Model 1	Model 2	Model 3	Model 4	Model 5
Controls					
CEO male	0.136	0.117	0.232	0.162	0.044
CEO age	−0.053	−0.053^*^	−0.071	−0.044	−0.038
CEO education	−0.061	−0.066	−0.031	−0.000	−0.047
CEO tenure	0.002	0.001	0.001	0.000	−0.000
Private firm	−0.231^*^	−0.227^*^	0.081	0.200	0.163
Firm size	−0.174	−0.157	−0.499^**^	−0.410^**^	−0.499^**^
Firm age	−0.003	−0.002	−0.013^*^	−0.012^*^	−0.011^*^
Independent					
CEO EO	0.242^***^	0.240^***^	0.217^***^	0.092	0.072
Mediator					
Dynamic Capabilities				0.514^***^	0.485^***^
Moderator					
Manufacturing		−0.083			−0.365^*^
Interaction					
CEO EO^*^ Manufacturing		0.143^*^			0.034
R^2^	0.369	0.390	0.219	0.292	0.314
R^2^ change	0.192	0.021	0.067	0.073	0.022

*N=188; CEO, Chief Executive Officer; and EO, Entrepreneurial Orientation.*

* *p<0.05;*

** *p<0.01;*

*** *p<0.001.*

According to Table 2, CEO entrepreneurial orientation positively resulted in firm performance (*b*=0.217, se=0.057, *t*=3.834, *p*<0.001; Model 3) and corporate dynamic capability (*b*=0.242, se=0.034, *t*=7.199, *p*<0.001; Model 1). According to Model 4, when both CEO entrepreneurial orientation and dynamic capability were added, the influence of CEO entrepreneurial orientation on firm performance was no longer significant. However, the influence of dynamic capability on firm performance remained significant (*b*=0.514, se=0.123, *t*=4.185, *p*<0.001), indicating that dynamic capability played a mediating role in the relationship between CEO entrepreneurial orientation and firm performance. Through the indirect effect test of 5,000 times of bootstrapping, the indirect effect of CEO entrepreneurial orientation on firm performance through dynamic capability was significant (indirect effect=0.124, se=0.041, 95%CI=[0.051, 0.210]). Thus, Hypotheses 1, 2, and 3 were supported.

**Figure 2:**
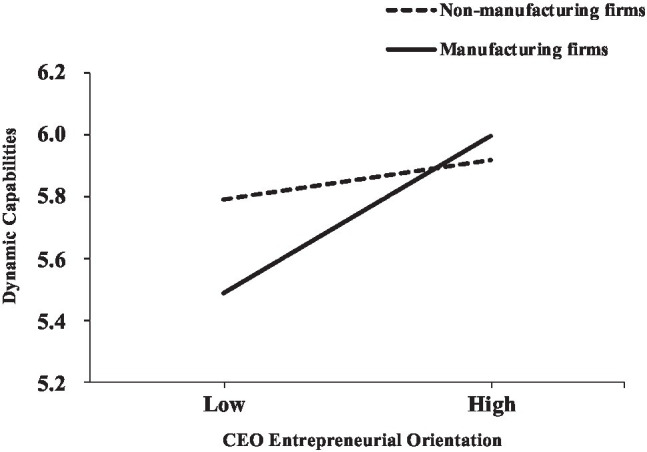
The moderating effect of firm industry type in the relationship between CEO entrepreneurial orientation and dynamic capabilities.

According to Model 2 in Table 2, the interaction between CEO entrepreneurial orientation and manufacturing was positively and significantly related to dynamic capability (*b*=0.143, se=0.064, *t*=2.219, *p*<0.05). The result shows that for manufacturing firms, the influence of CEO entrepreneurial orientation on dynamic capability was higher. Figure 2 shows the moderating effect of manufacturing firms. When a firm belonged to the manufacturing category (*b*=0.306, se=0.047, *t*=6.625, *p*<0.001), the influence of CEO entrepreneurial orientation on dynamic capability was stronger than that of non-manufacturing firms (*b*=0.164, se=0.048, *t*=3.505, *p*<0.001). Therefore, Hypothesis 4 was supported.

Table 3 shows the results of the moderating mediation effect. It shows that in manufacturing firms (*b*=0.149, se=0.049, 95% CI=[0.060, 0.251]), the direct effect of CEO entrepreneurial orientation on firm performance through dynamic capability was stronger than that in non-manufacturing firms (*b*=0.080, se=0.036, 95%CI=[0.023, 0.166]). The difference in the coefficients of indirect effects between manufacturing and non-manufacturing firms was significant (difference=0.069, se=0.037, 95%CI=[0.012, 0.160]), supporting Hypothesis 5.

**Table 3:** The results of moderated mediation.

Moderators	CEO EO→Dynamic Capabilities → Firm Performance		
	Indirect effect	SE	95%CI
Non-manufacturing	0.080	0.036	[0.023, 0.166]
Manufacturing	0.149	0.049	[0.060, 0.251]
Difference	0.069	0.039	[0.012, 0.160]

*CEO, Chief Executive Officer; EO, Entrepreneurial Orientation*.

## Discussion

From the perspective of dynamic capabilities, we explored the mediating mechanism of how CEO entrepreneurial orientation influences firm performance through dynamic capabilities and examined the moderating effect of firm industry type on this mediating mechanism. Using matched data from 188 Chinese firms, we found that CEO entrepreneurial orientation had a significant positive impact on dynamic capabilities and firm performance; dynamic capabilities mediated the relationship between CEO entrepreneurial orientation and firm performance; and firm industry type moderated the relationship between CEO entrepreneurial orientation and dynamic capabilities. In manufacturing enterprises, the indirect effect of CEO entrepreneurial orientation on firm performance through dynamic capabilities was stronger. These results have important theoretical and practical significance.

### Theoretical Implications

Our study makes several important contributions to the entrepreneurial orientation literature. First, it contributes to a small research stream in the existing entrepreneurial orientation literature that investigates the implications of CEO entrepreneurial orientation on firm performance (Chatterjee and Hambrick, 2007; Simsek et al., 2015; Keil et al., 2017; Liu and Xi, 2021). Most of the existing entrepreneurial orientation literature has treated entrepreneurial orientation as a firm-level construct and investigated the relationship between entrepreneurial orientation and firm performance (Higgs and Rowland, 2005; Covin et al., 2006; Rauch et al., 2009; Keil et al., 2017; Shen et al., 2021). However, the CEOs of firms are vital decision makers and facilitators of a firm’s entrepreneurial activities and are ultimately responsible for the organizational outcomes (Simsek et al., 2010, 2015; Bao et al., 2020). Therefore, understanding their roles in driving entrepreneurial orientation is important. By paying considerable and consistent attention to entrepreneurship, CEOs can shape firm-wide entrepreneurial orientation and behaviors. This consistent attention to entrepreneurship is called CEO entrepreneurial orientation (Keil et al., 2017; Liu and Xi, 2021). Although we are not the first to study it (Keil et al., 2017), we are among the first to investigate its implications for performance. Our findings suggest that CEO entrepreneurial orientation positively leads to firm performance by emphasizing value-creating entrepreneurial opportunities, encouraging organizational members’ innovative and pioneering behaviors, and investing efforts to anticipate demand and promote new products or services.

Second, our study helps understand of the role of dynamic capabilities in linking CEO entrepreneurial orientation and firm performance. Although only one study has investigated the linkages between CEO entrepreneurial orientation and firm value creation (Keil et al., 2017), providing a lens to support the fact that CEO entrepreneurial orientation is relevant to firm performance, the underlying mechanism has not been fully explored. Our paper is among the first to investigate the underlying mechanisms of CEO entrepreneurial orientation and firm performance. We found that CEO entrepreneurial orientation enhances the capabilities of organizational sensing and seizing of opportunities, managing threats and reconfiguration, that is, improves dynamic capabilities of the organization, and ultimately raises firm performance. This study integrates the literature on CEO entrepreneurial orientation and dynamic capabilities and provides a new perspective on the mediating mechanism in order to explore the relationship between CEO entrepreneurial orientation and firm performance.

Third, this study took an initiative to examine the moderating effect of firm industry type as a boundary condition for the “value” of CEO entrepreneurial orientation. Previous studies on manufacturing have mainly used data from manufacturing enterprises to study problems of interest (Mao and Wang, 2015; Cheng and Song, 2016; Yang et al., 2019), whereas a few have directly investigated the manufacturing industry and have treated it as a moderating variable. Against the backdrop of China’s manufacturing enterprises’ transformation, upgrading, and emphasis on innovation, and gradual loss of advantages in population and land cost (Jin, 2015; Cheng and Song, 2016), manufacturing enterprises must pay more attention to the improvement of innovation and dynamic capabilities in order to cope with increasing technological and market uncertainties. This study shows that when compared to non-manufacturing firms, CEO entrepreneurial orientation has a stronger impact on the dynamic capabilities of manufacturing enterprises. This study supports the idea that firm industry type can act as a moderator in the relationship between the variables of interest, just like firm ownership (Campbell et al., 2010; Zahra, 2012; Wu et al., 2014) and size (Ha-Brookshire, 2009; Chelliah et al., 2010). The finding provides a theoretical and empirical reference for future research intending to use manufacturing industry as a moderating variable.

### Practical Implications

Our study has important practical implications that highlight the benefits of CEO entrepreneurial orientation and dynamic capabilities. First, our findings suggest that the CEO’s attention, emphasis, and openness to entrepreneurial activities and behaviors matters for firm performance and dynamic capabilities and that boards of directors should, therefore, take CEO entrepreneurial orientation into account in the selection and succession processes. Second, our study found that dynamic capabilities are positively related to firm performance and mediate the relationship between CEO entrepreneurial orientation and firm performance. Thus, organizations should take steps to increase their dynamic capabilities. For example, as the representative of the organization, the CEO should pay greater attention to entrepreneurial activities and behaviors.

Our study has two important practical implications for manufacturing enterprises. First, it indicated that in manufacturing enterprises, improving CEO entrepreneurial orientation is more conducive to improving dynamic capabilities. This presents a new idea and transformation path for China’s manufacturing enterprises in the critical period of industrial upgrading and digital transformation (Lin et al., 2020a). That is, CEOs take the initiative to pay attention to entrepreneurial activities within the industry. Second, this study found that when compared to non-manufacturing enterprises, in manufacturing enterprises, CEO entrepreneurial orientation has a stronger, indirect effect on firm performance by improving dynamic capabilities. There are two ways in which manufacturing enterprises can improve their performance. Enterprises (1) must strive to improve the level of entrepreneurial orientation, and let the CEO pay consistent attention to entrepreneurial activities and behaviors and (2) can achieve sustained competitive advantage by building and forming dynamic capabilities.

### Limitations and Future Research Recommendations

Our study has a few limitations that provide intriguing opportunities for future research. First, the data we use do not allow us to determine the cause-effect relationship that is implied in the model (Figure 1). Causal sequences are difficult to ascertain in the investigation of the existing relationship. In this case, however, it is unlikely that dynamic capabilities influence CEO entrepreneurial orientation. The relationship between entrepreneurial orientation and firm performance has been widely supported in the literature. Thus, future research may rely on a longitudinal design to collect multi-wave data to test the cause-effect relationship.

Second, in this study, we focused on the role of dynamic capabilities in linking CEO entrepreneurial orientation and firm performance. However, the potential mediating mechanisms linking CEO entrepreneurial orientation and firm performance go far beyond dynamic capabilities. For example, organizational learning (Jiao et al., 2010; Zhao et al., 2011), entrepreneurial learning (Shen et al., 2021), human resource management system (Xi et al., 2021), absorptive capability (Zhai et al., 2018), and middle managers’ cognition (Liu and Xi, 2021) may play mediating roles in the linkage between CEO entrepreneurial orientation and firm performance.

Third, we tested the proposed model with a sample of Chinese manufacturing and high-tech companies located at four economic and technological development zones, which limits the generalizability of the findings. Future research should examine whether the findings can be replicated using other samples. Besides, different cultural dimensions, such as individualism – collectivism, may influence people’s behaviors (Qian and Miao, 2016). For example, in an individualistic culture, entrepreneurs are more welcome and encouraged and entrepreneurship is valued by society (Dubina and Ramos, 2016). Given that China’s national culture is more collectivist, it is worth examining whether our findings can be applied to and replicated in countries that have individualistic cultures.

Finally, this study mainly examined the performance implication of CEO entrepreneurial orientation and found the mediating role of dynamic capabilities. Future research should examine the low-level consequences of CEO entrepreneurial orientation. For example, Wales (2016) called for examinations of (CEO) entrepreneurial orientation as a prerequisite for individual-level outcomes, such as employee innovative behavior.

## Data Availability Statement

The raw data supporting the conclusions of this article will be made available by the authors, without undue reservation.

## Author Contributions

MX and XG planned the study and collected the data. YL and YJ wrote the manuscript. YL and MX analyzed the data and wrote the manuscript. All authors listed have made a substantial, direct, and intellectual contribution to the work, and approved it for publication.

## Funding

This work was supported by the Natural Science Foundation of China (71802106 and 71902112), the China Postdoctoral Science Foundation (224922), and MOE (Ministry of Education in China) Project of Humanities and Social Science (grant no. 18YJC630201).

## Conflict of Interest

The authors declare that the research was conducted in the absence of any commercial or financial relationships that could be construed as a potential conflict of interest.

## Publisher’s Note

All claims expressed in this article are solely those of the authors and do not necessarily represent those of their affiliated organizations, or those of the publisher, the editors and the reviewers. Any product that may be evaluated in this article, or claim that may be made by its manufacturer, is not guaranteed or endorsed by the publisher.
